# A shot in the genome: how accurately do shotgun 454 sequences represent a genome?

**DOI:** 10.1186/1756-0500-5-259

**Published:** 2012-05-28

**Authors:** Emese Meglécz, Nicolas Pech, André Gilles, Jean-François Martin, Michael G Gardner

**Affiliations:** 1 IMBE UMR 7263 CNRS, IRD, Equipe Evolution, Génome et Environnement, Aix-Marseille University, Centre Saint-Charles, Marseille Cedex 3, 13331, France; 2 Montpellier SupAgro, INRA, CIRAD, IRD, Centre de Biologie et de Gestion des Populations, Campus International de Baillarguet, CS30016, Montferrier-sur-Lez, 34988, France; 3 School of Biological Sciences, Flinders University, GPO Box 2100, Adelaide, 5001, South Australia; 4 Australian Centre for Evolutionary Biology and Biodiversity, School of Earth and Environmental Science, University of Adelaide, Adelaide, 5005, South Australia

## Abstract

**Background:**

Next generation sequencing (NGS) provides a valuable method to quickly obtain sequence information from non-model organisms at a genomic scale. In principle, if sequencing is not targeted for a genomic region or sequence type (e.g. coding region, microsatellites) NGS reads can be used as a genome snapshot and provide information on the different types of sequences in the genome. However, no study has ascertained if a typical 454 dataset of low coverage (1/4-1/8 of a PicoTiter plate leading to generally less than 0.1x of coverage) represents all parts of genomes equally.

**Findings:**

Partial genome shotgun sequencing of total DNA (without enrichment) on a 454 NGS platform was used to obtain reads of *Apis mellifera* (454 reads hereafter). These 454 reads were compared to the assembled chromosomes of this species in three different aspects: (i) dimer and trimer compositions, (ii) the distribution of mapped 454 sequences along the chromosomes and (iii) the numbers of different classes of microsatellites. Highly significant chi-square tests for all three types of analyses indicated that the 454 data is not a perfect random sample of the genome. Only the number of 454 reads mapped to each of the 16 chromosomes and the number of microsatellites pooled by motif (repeat unit) length was not significantly different from the expected values. However, a very strong correlation (correlation coefficients greater than 0.97) was observed between most of the 454 variables (the number of different dimers and trimers, the number of 454 reads mapped to each chromosome fragments of one Mb, the number of 454 reads mapped to each chromosome, the number of microsatellites of each class) and their corresponding genomic variables.

**Conclusions:**

The results of chi square tests suggest that 454 shotgun reads cannot be regarded as a perfect representation of the genome especially if the comparison is done on a finer scale (e.g. chromosome fragments instead of whole chromosomes). However, the high correlation between 454 and genome variables tested indicate that a high proportion of the variability of 454 variables is explained by their genomic counterparts. Therefore, we conclude that using 454 data to obtain information on the genome is biologically meaningful.

## Findings

To test the representativeness (i.e. the probability of sequencing any given part of the genome is proportional to its size) of 454 reads obtained by partial genome shotgun sequencing of total DNA without enrichment, 454 reads of the honey bee (*Apis mellifera*) were compared to its genome assembly Amel 4.0 [1]. Genome sequences refer to the assembled chromosomes of *A. mellifera* DH4 strain (a total of 217 Mb) downloaded from the genome section of Genbank. It is essential to note that we have used the genome assembly of *A. mellifera*, as a reference, but this is not a gold standard. Most genome assemblies are not complete and the uncompleted parts usually contain repetitive regions. Therefore it is difficult to know what the real composition of the genome is, especially for repetitive regions.

The total length of 454 reads were 10.4 Mb, which gives 0.048 fold coverage. This is undoubtedly very low for genome sequencing and assembly, but it is of typical order of magnitude for many non-model species, where the aim is not to sequence the whole genome but either to obtain a snapshot of the genome [2] or simply have sequence data to establish molecular markers [3,4]. The aim of this paper was to test how well a limited amount of DNA sequence can be used for estimating genome composition, therefore we did not eliminate any part of the genome such as repetitive or low complexity regions from the analyses. Although, in our case, a genome guided assembly would have been feasible, this is not the situation for non-model organisms. Since we are focusing on the suitability of 454 genome shotgun sequencing to obtain information on genome composition in non-model organism we did not use genome guided assembly. Additionally, we have chosen not to use *de novo* assembly of the 454 reads since, due to the low coverage and single end library, the assembly of repetitive region is particularly difficult. This can introduce a bias either by leaving the repetitive regions non-assembled, thus leading to overrepresentation of these regions in 454 reads or on the other extreme by eliminating some of the repetitive DNA that are highly similar, but represent different loci. Gomez-Alvarez et al. found that a non-negligible portion of the 454-based sequencing reads are artificial replicates in bacterial metagenomes [5]. This potential artefact of 454 data is likely to be due to amplified DNA attaching to empty beads during emulsion PCR creating multiple reads from a single template [6]. This artefact is very limited in our dataset, where only 1.48% of the reads appear to be part of a cluster, and the largest cluster contains only three reads (evaluated by 454 Replicate Filter [5]; cutoff 0.95, length requirement 0 and initial base pair match 5). Since duplicates are rarely removed in a typical 454 sequence analysis and the sequences that form clusters arise randomly [5], we did not eliminate potentially duplicated reads.

Three different types of comparisons were used in the analyses: (i) counts of observed di- and trinucleotide sequences in both the 454 data and in the complete genomes (variables: number of dimers and number of trimers), (ii) the distribution of the 454 sequence reads along the chromosomes (variables: number of 454 reads mapped to each chromosome fragments of one Mb, number of 454 reads mapped to each chromosome) and (iii) counts of different microsatellite classes as defined by their motif, repeat unit length and repeat number, and repeat unit length only (variables: number of microsatellites of each class). Dimer, trimer and microsatellite frequencies were chosen for the analyses due to their straightforward and unambiguous identification in the 454 data without the need of preliminary genome information like mRNA, protein or transposable element sequences.

## Laboratory protocols

DNA from *A. mellifera mellifera* from Nimes (France) was sheared by sonication. 1 μg of purified DNA was used for 454 FLX Titanium library (Roche Applied Science) preparation, according to the manufacturer’s protocols, at Genoscreen (Lille, France). Emulsion PCR (emPCR) was carried out at a ratio of 1 copy per bead, with subsequent disruption with isopropanol. Beads containing amplified DNA fragments were enriched and recovered for sequencing, to provide 50,000 to 70,000 enriched beads for each library. The recovered ssDNA beads were packed onto region 1/8 of a 70 mm × 75 mm Titanium PicoTiter plate and sequenced with 200 cycles. Sample preparation and analytical processing, such as base calling and filtering, were performed at Genoscreen (Lille, France), according to the manufacturer’s protocol for the Titanium series and Genome Sequencer FLX System Software (v2.3).

Reads were deposited to Short Read Archives of NCBI (SRS150264.1, 10.4 Mb of total length, 37870 reads).

## Statistical analyses

In order to establish the link between variables derived from 454 data and from their corresponding genomes, Pearson’s correlation tests were performed except when the variables deviated from normal distribution (as determined by a Shapiro-Wilk normality test). In these latter cases, Spearman’s rank correlation tests were used. When these tests resulted in significant correlation, we determined if the distribution of the variables were identical by performing chi-square tests. If the expected number of observations were greater than five in all classes, the *P*-value was obtained from *χ*2 distribution. Otherwise, a permutation test (N = 2000) was performed. To visualise the contribution of each element to the deviation between expected and observed values in the chi-square tests, barplots were created with the chi-square values of each element multiplied by the sign of the difference: negative for underrepresentation- and positive for over-representation. All statistical analyses were performed with R [7].

## Dimer and trimer composition

All observed 16 dimer and 64 trimer types were counted in the 454 data and in the genome in a sliding window of size two and three (e.g. ACCC is counted as 1 AC and 2 CC dimers or 1 ACC and 1 CCC trimer). The numbers of reverse complementary motifs were pooled (e.g. AG and CT are pooled under the label AG). We compared the counts of all possible dimer (10) and trimer (32) motifs of 454 data to genome counts of these motifs by chi-square tests and by Spearman’s rank correlation tests (Table 1).

**Table 1:** Results of correlation and Chi-square tests between 454 and genome variables

	**Correlation**			**Chi-square test**		
	**r**	df^*^	**P**	***χ*2**	df^*^	**P**
Number of dimers	0.988	NA	2.2e-16	9126	9	2.2e-16
Number of trimers	0.996	NA	2.2e-16	19275	31	2.2e-16
Number of dimers without AA/TT	0.999	7	1.8e-10	3567	8	2.2e-16
Number of trimers without AAA/TTT	0.996	NA	2.2e-16	10231	30	2.2e-16
Number of 454 reads per fragment	NA	NA	NA	635.9	225	2.2e-16
Number of 454 reads per chromosome	0.991	NA	3.2e-06	24.2	15	0.0624
Number of different microsatellite types (pooled by repeat unit length and repeat number)	0.972	21	9.3e-15	187.5	NA	0.0005
Number of microsatellites with di-trinucleotide motifs (pooled by motif)	0.969	NA	2.2e-16	77.9	13	2.713e-11
Number of different microsatellite types (pooled by repeat unit length)	1	NA	0.016	8.7	4	0.0685

^*^NA in df correspond to permutation tests.

**Figure 1:**
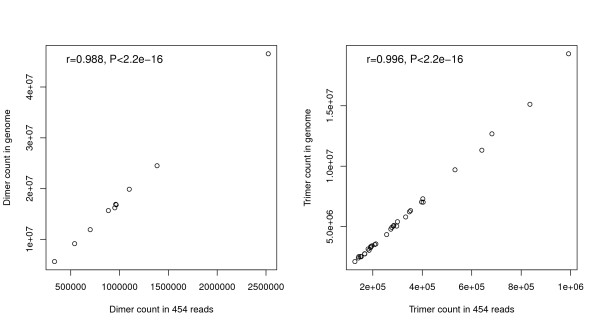
Correlation between the number different dimers and trimers in 454 and in genome data.

For both dimer and trimer counts, correlation coefficients were higher than 0.98 and correlation tests were highly significant between motif counts in the 454 run and in the genome (Figure 1; Table 1), indicating that variability of 454 shotgun data are mainly driven by the composition of the whole genome. The result of the chi-square test, however highlight a significant deviation between the motif count of 454 data and the genome (Table 1). The underrepresentation of AA/TT and AAA/TTT motifs contributed most to the deviation between the 454 and the genome data [see Figure S1 and S2 in Additional file 1. The deviations of other frequent motifs such as GA/TC, AAT/ATT, ATA/TAT are clearly less important. This deviation of homopolymer motifs may be the consequence of the problems associated with sequencing long homopolymers in 454 sequences [7]. Previous research has shown that homopolymer length is biased downwards in 454 reads especially for longer homopolymers [8], which leads to lower numbers of homodimers and homotrimers than expected. It is to note however, that CC/GG motifs were slightly over-represented in the 454 reads and the number CCC/GGG differed very little from the expected values. Since these motifs are rare in the genome compared to the other motifs, and thus the homopolymer length of G/C is much shorter (data not shown) it is not surprising that we did not detect the downward bias in the CC/GG, CCC/GGG motif frequency in 454 data. Deleting AA/TT and AAA/TTT motifs from the analyses, where the overrepresentation is likely to come from the biased homopolymer length of 454 sequences still yielded a significant deviation between 454 and genome data, however the chi-square values decreased considerably (Table 1).

Aird et al. have found that a PCR amplification step in Illumina sequencing introduces a considerable downward bias of regions with extreme A/T compositions [9]. Although the same phenomenon has not been studied in 454 sequencing, since it also includes a PCR amplification, it is possible that A/T or G/C rich regions are less well sequenced than the rest of the genome. This is of particular interest, since the genome of *A. mellifera* is particularly A/T rich [1]: 65% in mapped contigs and even higher in unmapped ones. Since a particular effort has been made to include extreme AT rich regions in the genome sequencing [1] the underrepresentation of A/T homopolymer motifs in the 454 reads could also come from this potential bias. However, the count of other A/T-rich non-homopolymer dimer and trimer motifs in the 454 reads do not deviate strongly from the genome counts [see Figures S1 and S2 in Additional file 1, thus the 454 specific homopolymer length bias is a more likely explanation for the underrepresentation of the A/T homopolymers.

At a genomic scale the very large number of observations renders chi-square tests extremely powerful to detect differences between 454 and genome distributions. It is undeniable that the 454 data does not reflect perfectly the genome compositions regarding dimer and trimer motifs, as evidenced by the significant results of the chi-square tests, however the very high correlation coefficient between 454 and genome data indicates that 0.97^2^ =0.94 of the variability of genome variables (here counts of dimer or trimer motifs) is explained by their 454 counterparts. Therefore, in spite of the statistically significant differences detected by the chi-square tests, we conclude that using 454 data to obtain information on the genome is biologically meaningful.

## Distribution of the 454 sequence reads along the chromosomes

The 37,870 reads obtained from a 454 run with a total length of 10.4 Mb were BLASTed (e = 1e-20, no dust filter, default parameters otherwise) to the genome (183.3 Mb without gaps). No filtering was done for low complexity sequences in order to be able to retain repetitive and low complexity sequences. For each 454 read the best hit was selected and accepted only if (i) the hit covered at least 80% of the 454 read and (ii) the identity over the aligned region was higher than 90%. When there were more than one hit with the same e-value a single hit was selected randomly. The best hit position on the chromosomes was recorded and the number of hits was counted for each Mb fragment of chromosome. These chromosome fragments did not overlap. If a 454 read was mapped on the junction of two fragments it was attributed to the first. Since the genome assembly contain a non-negligible proportion of gaps (Ns), for each chromosome fragment a number of the informative bases (not Ns) were counted and used to calculate the corrected number of hits for each Mb fragment (Number of 454 mapped into the fragment/Number of informative bases in fragment*1000000).

After the above described filtering, 29,862 reads (78.9% of the total number of reads) were mapped to the chromosomes. A possible explanation for this relatively low mapping success is a high proportion of gaps in the current *A. mellifera* assembly (16.1%).

**Figure 2:**
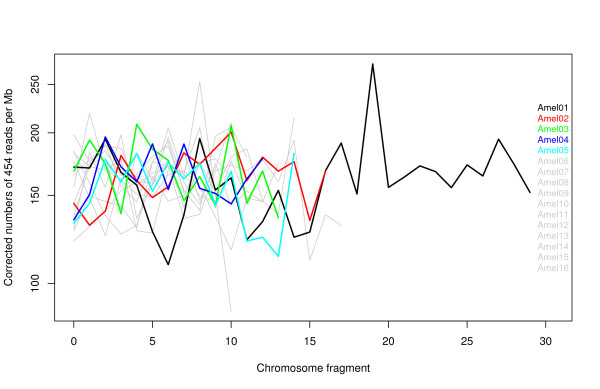
**Corrected number of 454 reads mapped to each one Mb chromosome fragment.** The number of 454 hits mapped to each Mb fragment of the genome (including Ns) is corrected for 1 Mb of informative bases (not Ns). Only the first five chromosomes are coloured.

**Figure 3:**
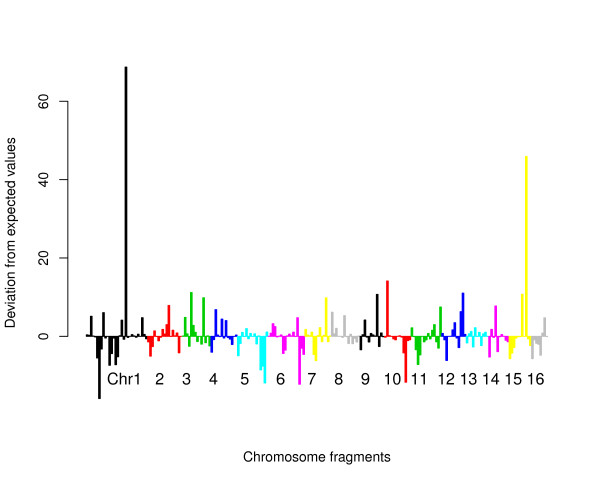
**Contribution of each chromosome fragment to the deviation between observed and expected number of mapped 454 reads.** Chi-square values of each element with the negative sign for under- and positive for over-representation. Successive chromosomes are marked by different colours.

Figure 2 indicates that the corrected number of hits for each chromosome varies considerably among fragments. A chi-square goodness of fit statistics also indicated that 454 reads were not distributed randomly along the chromosomes (Table1). Figure 3 shows the contribution of each fragment to the deviation from expected values. There are only two chromosome fragments that are strongly over-represented, but the chi-square test remains significant after deleting these fragments (*χ*2 = 523.4, df = 223, p < 2.2e-16), implying that the inter-fragment variation was not only due to these two fragments.

A possible explanation of the overrepresentation of a few fragments is that some repetitive regions are missing from the genome assembly. If only a few loci of a transposable element is included in the assembly, while many others are in the non-assembled part of the genome, 454 reads of these later loci can be erroneously mapped into the assembled loci, and cause overrepresentation of the region. Hiller et al. have shown that the overrepresentation of the only copy of an rDNA locus in *Caenorhabditis elegans* assembled genome is the result of the presence of several unmapped rDNA loci [10]. The genome of *A. mellifera* contains an unusually low number of transposons and retrotransposons (constituting only 1% of the assembled genome), most of which are members of the *Mariner* family [1]. Genomic screens for highly repetitive elements identified 3% of the genome, mostly mapping to telomeres and centromeres of the chromosomes by in situ mapping [11,12]. This implies that the apparent heterogeneity among the chromosome regions can be in fact due to technical artefact of mapping, and representativeness of the different regions are better in reality than we could expect it from our tests.

Although the dimer and trimer composition of the 454 reads does not seem to support bias in base composition in the 454 reads, we have tested if the base composition of each fragments (GC%) correlated with the corrected number of mapped hits. Spearman’s rank correlation test indicated a highly significant correlation (*P* = 1.12e-10), but a low correlation coefficient (0.415) indicates that a high proportion of the variability of the 454 read distribution cannot be explained by the G/C content of the fragment. Nevertheless the general trend is that fewer reads are mapped to fragments with low G/C content, which is likely to be due to amplification bias during the emulsion PCR step of sequencing [9]. A similar phenomenon was also observed in Solexa sequencing that also contains a PCR step before the actual sequencing [10].

At last, it is possible that the general background heterogeneity among chromosome fragment can be a result of the low coverage, and is thus due to chance events.

**Figure 4:**
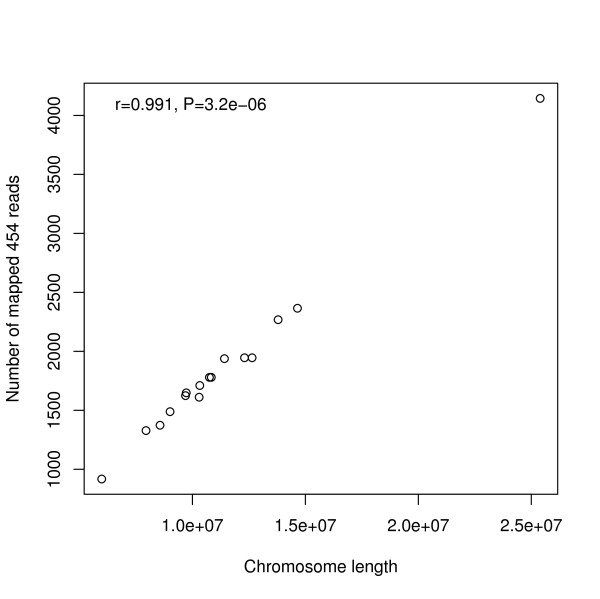
**Correlation between the number of hits mapped to each chromosome against the length of the chromosomes.** Only informative bases were counted for each chromosome length.

When pooling hit numbers by chromosomes, the chi-square test was not significant (Table 1). As discussed above, given the large number of observations at the genomic scale, the chi-square tests are very sensitive to detect differences. Thus the non-significant result of the chi-square test for the pooled data implies that the different chromosomes are uniformly represented by 454 data. This is further supported by a highly significant correlation observed between the chromosome length and the number of hits per chromosome (Figure 4; Table 1).

## Microsatellite composition

Microsatellites are commonly used as molecular markers, thus are of interest to the scientific community. *Apis mellifera* has a large number of microsatellites compared to other insects [13], and indeed linkage map based on microsatellites provided valuable assistance in the genome assembly [14].

**Figure 5:**
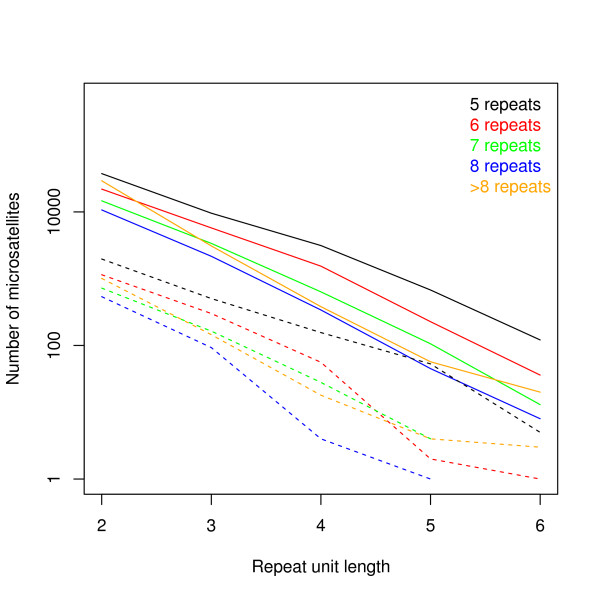
**Number of different microsatellite types in 454 and genome sequences.** Microsatellite count in the genome (continuous lines) and 454 data (dashed lines).

All perfect microsatellites of 2–6 bp motifs with at least 5 repetitions were counted in the genome and in the 454 data set. The number of microsatellites decreased generally with increasing repeat unit length and repeat number both in the genome and in the 454 data (Figure 5). Microsatellite counts were compared between the genome and the 454 reads by pooling their numbers in three different ways: by (i) individual motifs for di and trinucleotide motifs, (ii) repeat unit length (di-hexanucleotides) and repeat number (5, 6, 7, 8, >8) and (iii) repeat unit length only.

Pearson’s correlation tests were performed on the number of microsatellites of different classes between the 454 data and its corresponding genome, and a highly significant correlation was detected in all three kinds of pooling (Table 1) [see Figures S3 and S4 in Additional file 1]. However, by pooling microsatellites by motif or by repeat unit length and repeat number, the chi-square tests still showed significant differences of the microsatellite class distribution between the 454 data and the corresponding genome (Table 1). Most of the deviation came from the under-representation of long (>8 repeats) microsatellites with a dinucleotide motif when pooling by repeat unit length and repeat number [see Figure S5 in Additional file 1], and by the overrepresentation of the AAC motif when pooling by motif [see Figure S6 in Additional file 1]. Thus, the possible downwards bias of A/T rich regions suggested by the analysis of the mapped 454 read distribution is not detected in the microsatellite composition.

By pooling microsatellite numbers by repeat unit length, the chi-square test became non-significant (*χ*2 = 8.7, df = 4, *P* = 0.0685). This indicates that, although 454 data does not reflect perfectly the microsatellite composition of the genome if microsatellites are pooled at a fine scale, by pooling data into larger categories, 454 data gives a good approximation of the genome content.

## Conclusions

Next generation sequencing has increased the quantity of sequences by several orders of magnitudes compared to pre-genomics area. This high number of observations increases the power of statistical tests which become very sensitive to detect small differences among most of the studied variables. What does this mean practically to a biologist? Where do the differences between genome assembly and 454 shotgun come from? There are five potential causes: (i) the low coverage of the 454 reads increases the probability that 454 reads differ from the genome assembly by chance alone; (ii) The assembled genome is a DH4 strain of primarily *A.m. ligustica*[15], while we have 454 reads of *A.m.mellifera*. These subspecies are two of the most distantly related among all subspecies of *A. mellifera*[16]. The genetic variation between these subspecies may be the cause of some of the observed differences between the genome and the 454 data, but is unlikely to introduce a systematic bias; (iii) The emulsion PCR has been shown to amplify sequences with extreme C/G content less efficiently [9]. This can explain that A/T rich chromosome fragments are generally less well covered by 454 reads in our analyses. This reduced PCR efficiency could also lead to the underrepresentation of A/T rich motifs (both in microsatellites and genome-wise counts), but since it is not detected in our analyses this source of error is unlikely to be important; (iv) Homopolymer length bias characteristic of 454 sequencing is likely to be the reason of A/T homodimer and homotrimer bias observed in our analyses; (v) The incomplete genome assembly leads to a possibility of erroneously mapping repetitive elements to the assembled region, which thus appear to be overrepresented. Therefore, in this respect 454 shotgun sequences might provide a better estimation of repetitive DNA content than the genome assembly.

Can we use 454 data to infer information on the genome composition? No doubt that the 0.05x coverage 454 data is not a random sample of the assembled part of the genome. However, the very strong correlation between 454 and genome variables and a lack of significant deviation for pooled data implies that 454 sequences provide a useful approximation of the genomic content. Furthermore, increasing the genome coverage is likely to reduce random sampling errors, thus could provide an ever better estimation.

## Competing interests

The authors declare that they have no competing interests.

## Authors' contributions

EM conceived and performed the bioinformatics and statistical analyses and wrote the paper. NP conceived the statistical analyses and helped in the interpretation of the results. AG and MGG helped the design of the analyses. JFM provided 454 data. All authors read and significantly participated in the writing of the paper, and approved the final manuscript.

## Supplementary Material

Additional file 1 **Figure S1.** Contribution of each dimer motif to the deviation between 454 and genome motif counts. Chi-square values of each element with the negative sign for under- and positive for over-representation. Figure S2 Contribution of each trimer motif to the deviation between 454 and genome motif counts. Chi-square values of each element with the negative sign for under- and positive for over-representation. Figure S3 Correlation between the number of different microsatellites pooled by repeat unit length and repeat number in the genome against their numbers in 454 reads. Figure S4 Correlation between the number of different microsatellites pooled by motif in the genome against their numbers in 454 reads. Figure S5 Contribution of each microsatellite type to the deviation between 454 and genome microsatellite counts. Chi-square values of each element with the negative sign for under- and positive for over-representation. Figure S6 Contribution of each microsatellite motif to the deviation between 454 and genome microsatellite counts. Chi-square values of each element with the negative sign for under- and positive for over-representation. Click here for file

## References

[B1] The Honeybee Genome Sequencing Consortium Insights into social insects from the genome of the honeybee Apis mellifera Nature 2006 443 931 949 10.1038/nature05260 17073008 PMC2048586

[B2] Rasmussen DA Noor MA What can you do with 0.1× genome coverage? A case study based on a genome survey of the scuttle fly Megaselia scalaris (Phoridae) BMC Genomics 2009 10 382 10.1186/1471-2164-10-382 19689807 PMC2735751

[B3] Malausa T Gilles A Meglécz E Blanquart H Duthoy S Costedoat C Dubut V Pech N Castagnone-Sereno P DéLye C Feau N Frey P Gauthier P Guillemaud T Hazard L Le Corre V Lung-Escarmant B Malé P-JG Ferreira S Martin J-F High-throughput microsatellite isolation through 454 GS-FLX Titanium pyrosequencing of enriched DNA libraries Mol Ecol Resour 2011 11 638 644 10.1111/j.1755-0998.2011.02992.x 21676194

[B4] Gardner MG Fitch AJ Bertozzi T Lowe AJ Rise of the machines - recommendations for ecologists when using next generation sequencing for microsatellite development Mol Ecol Resour 2011 11 1093 1101 10.1111/j.1755-0998.2011.03037.x 21679314

[B5] Gomez-Alvarez V Teal TK Schmidt TM Systematic artifacts in metagenomes from complex microbial communities ISME J 2009 3 1314 1317 10.1038/ismej.2009.72 19587772

[B6] Briggs AW Stenzel U Johnson PLF Green RE Kelso J Prufer K Meyer M Krause J Ronan MT Lachmann M Paabo S Patterns of damage in genomic DNA sequences from a Neandertal Proc Natl Acad Sci 2007 104 14616 14621 10.1073/pnas.0704665104 17715061 PMC1976210

[B7] Gilles A Meglécz E Pech N Ferreira S Malausa T Martin J-F Accuracy and quality assessment of 454 GS-FLX Titanium pyrosequencing BMC Genomics 2011 12 245 10.1186/1471-2164-12-245 21592414 PMC3116506

[B8] Margulies M Egholm M Altman WE Attiya S Bader JS Bemben LA Berka J Braverman MS Chen Y-J Chen Z Dewell SB Du L Fierro JM Gomes XV Godwin BC He W Helgesen S Ho CH Ho CH Irzyk GP Jando SC Alenquer MLI Jarvie TP Jirage KB Kim J-B Knight JR Lanza JR Leamon JH Lefkowitz SM Lei M Li J Lohman KL Lu H Makhijani VB McDade KE McKenna MP Myers EW Nickerson E Nobile JR Plant R Puc BP Ronan MT Roth GT Sarkis GJ Simons JF Simpson JW Srinivasan M Tartaro KR Tomasz A Vogt KA Volkmer GA Wang SH Wang Y Weiner MP Yu P Begley RF Rothberg JM Genome sequencing in microfabricated high-density picolitre reactors Nature 2005 437 376 380 16056220 10.1038/nature03959 PMC1464427

[B9] Aird D Ross MG Chen W-S Danielsson M Fennell T Russ C Jaffe DB Nusbaum C Gnirke A Analyzing and minimizing PCR amplification bias in Illumina sequencing libraries Genome Biol 2011 12 R18 10.1186/gb-2011-12-2-r18 21338519 PMC3188800

[B10] Hillier LW Marth GT Quinlan AR Dooling D Fewell G Barnett D Fox P Glasscock JI Hickenbotham M Huang W Magrini VJ Richt RJ Sander SN Stewart DA Stromberg M Tsung EF Wylie T Schedl T Wilson RK Mardis ER Whole-genome sequencing and variant discovery in C. elegans Nat Meth 2008 5 183 188 10.1038/nmeth.1179 18204455

[B11] Beye M Moritz RFA Characterization of Honeybee (Apis mellifera L.) Chromosomes Using Repetitive DNA Probes and Fluorescence in situ Hybridization J Hered 1995 86 145 150 7751599 10.1093/oxfordjournals.jhered.a111545

[B12] Sahara K Marec F Traut W TTAGG telomeric repeats in chromosomes of some insects and other arthropods Chromosome Res 1999 7 449 460 10.1023/A:1009297729547 10560968

[B13] Pannebakker BA Niehuis O Hedley A Gadau J Shuker DM The distribution of microsatellites in the Nasonia parasitoid wasp genome Insect Mol Biol 2010 19 91 98 20167020 10.1111/j.1365-2583.2009.00915.x

[B14] Solignac M Zhang L Mougel F Li B Vautrin D Monnerot M Cornuet J-M Worley KC Weinstock GM Gibbs RA The genome of Apis mellifera: dialog between linkage mapping and sequence assembly Genome Biol 2007 8 403 10.1186/gb-2007-8-3-403 17381825 PMC1868943

[B15] Zayed A Whitfield CW A genome-wide signature of positive selection in ancient and recent invasive expansions of the honey bee Apis mellifera Proc Natl Acad Sci USA 2008 105 3421 3426 10.1073/pnas.0800107105 18299560 PMC2265178

[B16] Whitfield CW Behura SK Berlocher SH Clark AG Johnston JS Sheppard WS Smith DR Suarez AV Weaver D Tsutsui ND Thrice Out of Africa: Ancient and Recent Expansions of the Honey Bee, Apis mellifera Science 2006 314 642 645 10.1126/science.1132772 17068261

