# Supplementary material for: A shot in the genome: how accurately do shotgun 454 sequences represent a genome?
Source: BMC Res Notes. 2012 May 28;5:259. doi: 10.1186/1756-0500-5-259 (PMC3444912; doi:10.1186/1756-0500-5-259)
Supplement: Additional file 1 — Figure S1. Contribution of each dimer motif to the deviation between 454 and genome motif counts. Chi-square values of each element with the negative sign for under- and positive for over-representation. Figure S2 Contribution of each trimer motif to the deviation between 454 and genome motif counts. Chi-square values of each element with the negative sign for under- and positive for over-representation. Figure S3 Correlation between the number of different microsatellites pooled by repeat unit length and repeat number in the genome against their numbers in 454 reads. Figure S4 Correlation between the number of different microsatellites pooled by motif in the genome against their numbers in 454 reads. Figure S5 Contribution of each microsatellite type to the deviation between 454 and genome microsatellite counts. Chi-square values of each element with the negative sign for under- and positive for over-representation. Figure S6 Contribution of each microsatellite motif to the deviation between 454 and genome microsatellite counts. Chi-square values of each element with the negative sign for under- and positive for over-representation. [file 1756-0500-5-259-S1.doc]

## Additional file 1

File format: PDF

#### Title: Supplementary graphics

Description: 3 supplementary figures

## Figure S1 - Contribution of each dimer motif to the deviation between 454 and genome motif counts.

Chi-square values of each element with the negative sign for under- and positive for over-representation.

## Figure S2 - Contribution of each trimer motif to the deviation between 454 and genome motif counts.

Chi-square values of each element with the negative sign for under- and positive for over-representation.

## Figure S3 - Correlation between the number of different microsatellites pooled by repeat unit length and repeat number in the genome against their numbers in 454 reads.

## Figure S4 - Correlation between the number of different microsatellites pooled by motif in the genome against their numbers in 454 reads.

## Figure S5 - Contribution of each microsatellite type to the deviation between 454 and genome microsatellite counts.

Chi-square values of each element with the negative sign for under- and positive for over-representation.

## Figure S6 - Contribution of each microsatellite motif to the deviation between 454 and genome microsatellite counts.

Chi-square values of each element with the negative sign for under- and positive for over-representation.
